# Aptamer Display on Diverse DNA Polyhedron Supports

**DOI:** 10.3390/molecules23071695

**Published:** 2018-07-11

**Authors:** Simon Chi-Chin Shiu, Lewis A. Fraser, Yifan Ding, Julian A. Tanner

**Affiliations:** School of Biomedical Sciences, Li Ka Shing Faculty of Medicine, The University of Hong Kong, Pokfulam, Hong Kong, China; simon.chichin.shiu@gmail.com (S.C.-C.S.); lewis-fraser@hku.hk (L.A.F.); yifand16@gmail.com (Y.D.)

**Keywords:** DNA nanostructure, aptamer, malaria, diagnostics, structural optimization

## Abstract

DNA aptamers are important tools for molecular recognition, particularly for a new generation of tools for biomedicine based on nucleic acid nanostructures. Here, we investigated the relative abilities of different shapes and sizes of DNA polyhedra to display an aptamer which binds to the malaria biomarker *Plasmodium falciparum* lactate dehydrogenase (PfLDH). The aptamer was shown to perform an Aptamer-Tethered Enzyme Capture (APTEC) assay with the hypothesis that the display of the aptamer above the surface through the use of a polyhedron may lead to better sensitivity than use of the aptamer alone. We compared different numbers of points of contact, different shapes, including tetrahedron, square, and pentagon-based pyramids, as well as prisms. We also investigated the optimal height of display of the structure. Our results demonstrated that the display of an aptamer on an optimized nanostructure improved sensitivity up to 6-fold relative to the aptamer alone in the APTEC assay. Other important factors included multiple basal points of contact with the surface, a tetrahedron proved superior to the more complex shaped structures, and height above the surface only made minor differences to efficacy. The display of an aptamer on a nanostructure may be beneficial for higher sensitivity aptamer-mediated malaria diagnosis. Aptamer displays using DNA nanostructure polyhedron supports could be a useful approach in a variety of applications.

## 1. Introduction

Aptamers are single-stranded oligonucleotides that can fold into specific secondary structures to facilitate molecular recognition events in diagnostics and therapeutics [1,2]. We previously identified a DNA aptamer against the malaria biomarker *Plasmodium falciparum* lactate dehydrogenase (PfLDH), which is useful in malaria point-of-care diagnostics [3,4]. The aptamer was found to be able to bind PfLDH specifically and differentiate it from human lactate dehydrogenase (hLDH) by identifying a loop in PfLDH that is missing in hLDH [5]. We have integrated the aptamer into a range of diagnostic strategies, including colorimetric assay [6,7], electrochemical sensing [8], and even incorporating it into DNA nanostructures, microfluidic devices, and a 3-D printed syringe to demonstrate the versatility of the aptamer [9,10,11,12,13]. Among all the applications of the PfLDH aptamer, the Aptamer-Tethered Enzyme Capture (APTEC) assay uses the intrinsic enzymatic activity of PfLDH to trigger a color change of nitrotetrazolium blue from colorless to blue as an observable signal [6]. While the APTEC assay is sensitive enough to diagnose malaria in most clinical blood samples of lower than 0.01% parasitaemia with 90% accuracy, it was not able to detect the disease in samples at <0.001% parasitaemia. Therefore, further improvement in sensitivity is important for a more robust diagnosis of malaria [12].

The APTEC assay was performed using a blood sample with the aptamer coated on a surface picking up the PfLDH from the sample. One possible factor affecting the sensitivity was the accessibility of the aptamer towards PfLDH in the bulk solution or blood sample [14,15]. We hypothesized that a DNA nanostructure could be conjugated to the aptamer as a support to increase the distance between the aptamer and the surface. We also considered that the rich negative nucleic acid nanostructure could facilitate the aptamer pointing up away from the surface to facilitate a stronger interaction with the protein target. We therefore asked the question of which shape and design of aptamer nanostructure would best improve the sensitivity of the APTEC assay.

**Figure 1:**
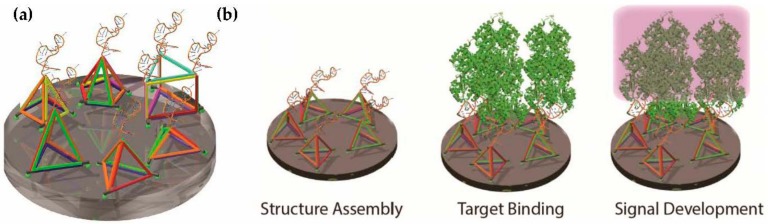
Polyhedra tested in this study. Different polyhedra were designed in this study to display the PfLDH aptamer on a streptavidin plate. (**a**) Designs included tetrahedra of different heights, square-based and pentagon-based pyramids, and prisms. The 5′-end at each vertex of the polyhedron was labelled with biotin (green spheres at the bottom vertexes) to allow attachment to the streptavidin surface. (**b**) Process of structure assembly, target binding, and signal development for the APTEC assay.

The DNA tetrahedron is well established as a simple three-dimensional nanostructure for detecting molecular targets [14,15,16,17]. As the tetrahedron was the only polyhedron nucleic acid nanostructure apparent in the literature, here, we investigated the relationship between different shapes beyond the tetrahedron, including square and pentagon-based pyramids, prisms, and tetrahedra of different heights and different numbers of points of attachment (Figure 1a). The entire process included structure assembly on the surface, protein target binding, and then APTEC signal development (Figure 1b). This experimental strategy provided a facile approach to perform a comparative characterization of the various polyhedra tested.

## 2. Results

### 2.1. Design and Assembly of Aptamer-Integrated DNA Nanostructures

**Figure 2:**
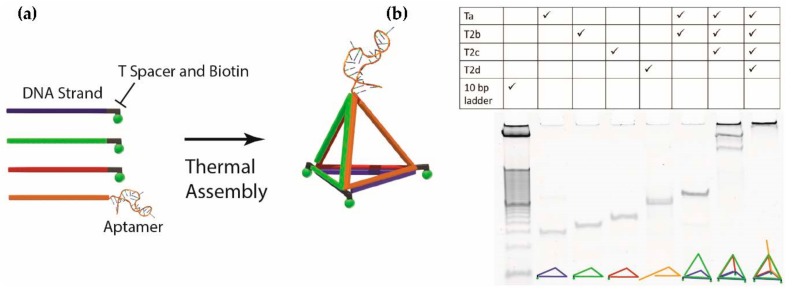
Assembly of an aptamer-tetrahedron. (**a**) Schematic diagram of DNA polyhedron assembly. Single-stranded DNAs were assembled into a specific nanostructure through thermal annealing from high temperature to room temperature. (**b**) Formation of an aptamer-tetrahedron on polyacrylamide gel. Each lane was loaded with 150 nM of a single-stranded DNA combination. The rightmost lane shows the assembled product. The multiple bands on the second last lane represent the multimer formed from the interaction between the exposed single-stranded regions on an incomplete tetrahedron.

In this study, eight different DNA nanostructures were investigated, including three tetrahedra of different heights, a square-based and pentagon-based pyramid, and three prisms exposing one to three PfLDH aptamers. The design and sequence generation were performed in Tiamat [18]. Before generating the sequence for the random region, a constant sequence was assigned to the structure to ensure the generation of specific duplex sequences for the assembly, including the sequence of PfLDH aptamer, the thymidine residues at each turning point of the nanostructure and the spacer at each base vertex. The assembly of these nanostructures was observed by polyacrylamide gel electrophoresis (PAGE). The formation of the tetrahedra (tetrahedron 2) is shown in Figure 2 and others are in the supplementary materials (Figure S1). In general, as shown in the gel image (Figure 2b), single-stranded DNAs were assembled as expected as the size increased according to the number of strands. Among all the gel images showing the assembly of nanostructures, we realized that there were some unexpected bands when the nanostructure was not completed. For example, we think the unexpected bands on the second last lane in Figure 2b represented random multimers of the incomplete tetrahedron because of the interaction between the exposed single-stranded regions.

### 2.2. Position of Aptamer-Tetrahedron on Surface and Comparison with Single-Stranded Aptamer

**Figure 3:**
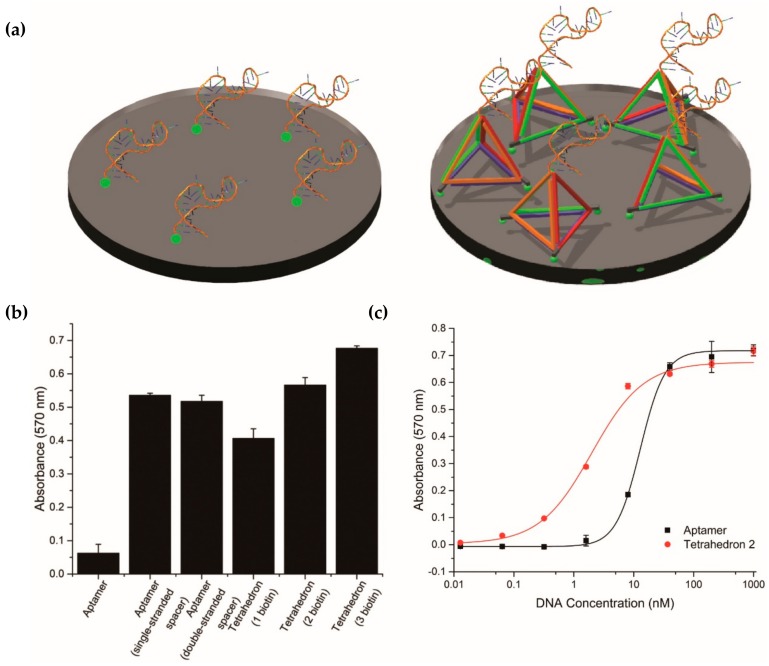
Comparison of aptamer-tethered enzyme capture (APTEC) assay using aptamer displayed directly alone versus on a DNA tetrahedron. (**a**) Design of the experimental setup. Comparison was done by using 10 nM of a single-stranded aptamer with different nanostructure supports, including single-stranded, doubled-stranded spacer, and a tetrahedron with an increasing number of biotin modifications at the 5′-ends. (**b**) Absorbance for different controls in APTEC as mentioned in (**a**). The signal intensity of an aptamer on a tetrahedron with three biotin labels performed the best. (**c**) The binding affinities (*K*_D_) of the aptamer and tetrahedron 2 toward PfLDH were 12.66 ± 0.71 nM and 2.09 ± 0.15 nM, respectively. The concentration of PfLDH was 100 ng/mL (2.7 nM).

After assembling the aptamer-tetrahedron, we coated it on a streptavidin surface to compare its performance with a single-stranded aptamer. Before that, we confirmed the three biotins on each vertex were coated on the surface successfully by assembling tetrahedra with one to three biotins as in Figure 3a and compared them to a single-stranded aptamer with a single and double-stranded spacer in the APTEC assay. As shown in Figure 3b, the result showed that all the samples with spacers or tetrahedra produced a higher absorbance in the APTEC assay than just the single-stranded aptamer alone. The signal intensity from the tetrahedron gradually increased, correlating with the increase in the number of biotin modifications’ tethers. Next, we determined the binding affinities of the tetrahedron-displayed and single-stranded aptamer. The fitting of a logistic equation found that the dissociation constant (*K*_D_) of the tetrahedron and single-stranded aptamer were 2.09 ± 0.15 nM and 12.66 ± 0.71 nM, respectively (Figure 3c). This result showed a clear benefit for assay sensitivity when the aptamer is displayed on the tetrahedron support relative to the aptamer alone. It also correlates to the hypothesis in the literature that tethering an aptamer on top of a tetrahedron would increase its accessibility to the analyte [14,15].

### 2.3. Investigation of Different Heights of Tetrahedron

**Figure 4:**
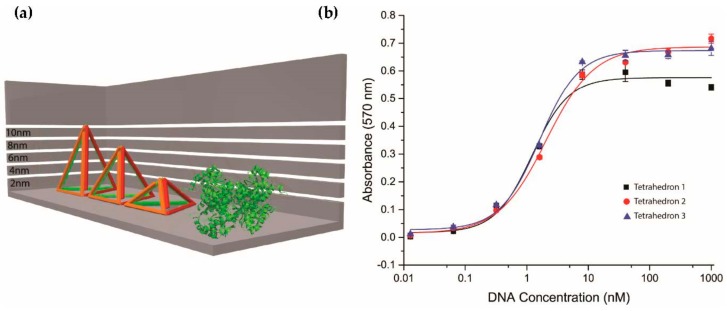
Comparison between DNA tetrahedra of different heights in APTEC. (**a**) Structures of the three DNA tetrahedra and PfLDH. The theoretical heights of DNA tetrahedra were 2.65 nm, 6.72 nm, and 10.55 nm. (**b**) The binding affinities (*K*_D_) of tetrahedra 1, 2, and 3 toward PfLDH were 1.22 ± 0.13 nM, 2.09 ± 0.15 nM, and 1.61 ± 0.10 nM respectively. The concentration of PfLDH was 100 ng/mL (2.7 nM).

Having established that the tetrahedron-displayed aptamer was superior to the aptamer alone in the APTEC assay, we next investigated various heights of the tetrahedra. We assembled tetrahedra of different heights (2.65 nm, 6.72 nm, and 10.55 nm), as in Figure 4a, and compared their affinity towards PfLDH in APTEC. Although the saturation intensity increased with height as observed in Figure 4b, the *K*_D_ values of these height variants were found to be similar (tetrahedron 1 = 1.22 ± 0.13 nM; tetrahedron 2 = 2.09 ± 0.15 nM, and tetrahedron 3 = 1.61 ± 0.10 nM). While the orientation of PfLDH on the tetrahedron remained unchanged with the increase in height, resulting in a similar binding affinity, it suggested the necessity of having a rigid structure to lift the aptamer from a surface and the increase in distance could help the captured PfLDH to be more accessible to other reagents in the APTEC assay.

### 2.4. Investigation of Tetrahedron Compared to Other Three-Dimensional Nanostructure Polyhedra

**Figure 5:**
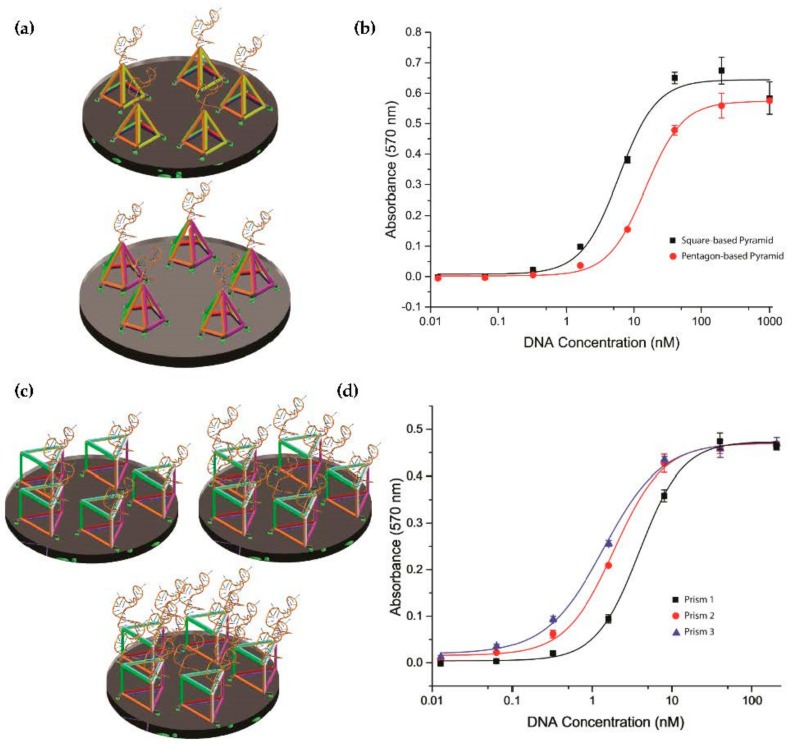
Performance of different polyhedron designs. (**a**) Structures of square-based, pentagon-based DNA pyramids, and prisms exposing different numbers of aptamers. Further points of contact were introduced in the pyramids to support the aptamer on the surface. (**b**) The binding affinities (*K*_D_) of square-based and pentagon-based DNA pyramid toward PfLDH were 5.99 ± 0.64 nM and 14.85 ± 0.80 nM, respectively. (**c**) Structure of DNA prisms exposing one to three PfLDH aptamers. (**d**) The *K*_D_ of a prism with one, two, and three aptamers toward PfLDH were 3.91 ± 0.18 nM, 1.89 ± 0.09 nM, and 1.38 ± 0.08 nM, respectively. The concentration of PfLDH was 100 ng/mL (2.7 nM).

The tetrahedron is the most commonly used DNA nanostructure for increasing the accessibility of sensing probes on surfaces to analytes due to its simple three-dimensional structure. We were interested in investigating more complex geometries of polyhedra to determine whether any showed benefits relative to the tetrahedron in aptamer display. Therefore, we first designed and assembled a square-based and pentagon-based pyramid as shown in Figure 5a. The difference between these two designs and the tetrahedron was the increase in the number of contact points between the nanostructures and the surface. The result in Figure 5b looking at the affinity showed that the *K*_D_ of the square-based and pentagon-based pyramid were 5.99 ± 0.64 nM and 14.85 ± 0.80 nM, respectively. As these affinities were weaker than for the tetrahedra in Figure 4, we concluded that the square-based and pentagon-based pyramids were less effective than tetrahedra for aptamer display. We then assembled a DNA prism, as in Figure 5c, exposing one to three PfLDH aptamers on its upper surface. The affinity study in APTEC (Figure 5d) found the *K*_D_ values of exposing one, two, and three aptamers were 3.91 ± 0.18 nM, 1.89 ± 0.09 nM, and 1.38 ± 0.08 nM, respectively. These results showed that although the prism did perform better than the single-stranded aptamer alone, the tetrahedron still appeared to be the simplest and most effective way to display the aptamer. One possible explanation may be that the increased complexity of the nanostructure reduced the yield of the nanostructure. As observed in Figure S1, there was some smearing on the lane of assembled product of pyramids and prisms that could indicate a lower yield of product relative to the tetrahedron assemblies. We concluded that the tetrahedron was the most effective polyhedron for supporting an aptamer on a surface to access PfLDH in bulk solution.

### 2.5. Detection of Exogenous Recombinant PfLDH in Whole Rat Blood

**Figure 6:**
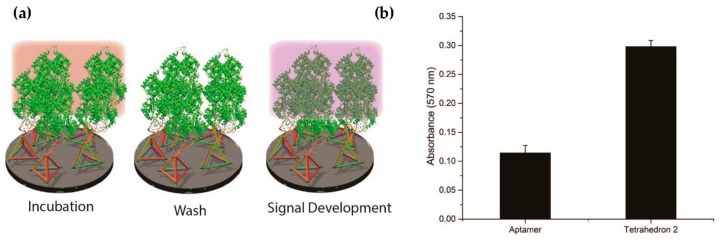
Detection of exogenous recombinant PfLDH in whole rat blood using a DNA tetrahedron. (**a**) Rat blood spiked with recombinant PfLDH was used during the incubation process to mimic a clinical condition. (**b**) The absorbance intensity of a 10 nM DNA tetrahedron in APTEC was higher than that of a 10 nM single aptamer. The concentration of PfLDH was 100 ng/mL (2.7 nM).

After confirming the tetrahedron to be the best among all other DNA nanostructures, we mimicked a clinical blood sample by spiking PfLDH into whole rat blood (Figure 6a). Similar to our previous observations using purified components, display of the aptamer on the tetrahedron provided a significantly increased APTEC assay response relative to the aptamer alone (Figure 6b).

## 3. Discussion

Our results in this particular assay showed that the tetrahedron is superior to the square-based pyramid, pentagon-based pyramid, and prism for the display of a nucleic acid aptamer. To our knowledge, this is the first time that these more complex geometries of nucleic acid nanostructure have been investigated for aptamer display. There may be circumstances where other geometries of polyhedron could prove better. The DNA tetrahedron is widely used in electrochemical sensing together with aptamers for detecting different analytes, including nucleic acids, proteins, and small molecules. Generally, DNA tetrahedra have incorporated strands which are modified with thiol labels at the bottom vertices for coating on a gold electrode. The sensing probe at the top vertex is usually coupled with other labeling techniques for signal generation, including avidin-horseradish peroxidase [19,20,21,22,23,24], silver or gold nanoparticles [25,26], fluorescence labels [27], and ferrocene [28]. The use of a tetrahedron in these scenarios increased accessibility of the sensing probe to other reagents for the signal generation. As the tetrahedron is rigid, strand-displacement was performed on the edge of the tetrahedron to detect the nucleic acid target, which led to an increase in distance between ferrocene label and the gold electrode [29]. An alternative approach has been used to determine pH by using a cytosine-rich i-motif on the edge of a tetrahedron sensitive to hydrogen ion concentration [30].

An aptamer accessible surface density is another factor that may influence the sensitivity of detection besides spatial accessibility. There are a few strategies using the conjugation of more than one tetrahedron to provide multiple valency for the incorporation of aptamers. By probing different sites on a nucleic acid target, two tetrahedra have been connected in an approach similar to the use of a secondary antibody leading to signal enhancement [31]. Since the tetrahedron is a three-dimensional structure that has multiple edges, multiple sites of strand displacement can also be integrated for nucleic-acid-based logic gates [32]. A similar concept could be extended whereby multiple tetrahedra come together upon the presence of a single target to achieve signal amplification [33]. We observed that a prism exposing multiple aptamers could achieve a higher sensitivity than a prism with a single aptamer (Figure 5d). Therefore, there may be value in approaches exposing multiple sensing probes as a simple alternative to conjugating multiple nanostructures.

In summary, we investigated different designs of DNA nanostructure as a support to elevate and display aptamers above a surface for binding to a target protein. Results showed that at least three points of contact were optimal for support, and simple tetrahedron support designs proved most effective. Results were consistent for spiked samples using rat blood. The display of aptamers on tetrahedron nanostructures may be an effective approach to generally increase the sensitivity of aptamer-enabled diagnostic devices.

## 4. Materials and Methods

### 4.1. Oligonucleotides, Enzymes and Other Chemicals

All oligonucleotides stated in Table S1 were purchased in lyophilized powder form from Integrated DNA Technologies (Coralville, IA, USA) with standard desalting. Single stranded DNA was dissolved in Milli-Q water to 100 µM. Recombinant PfLDH was expressed in *E. coli* BL21 (DE3) pLysS and purified by HisTrap chromatography (GE Healthcare, Chicago, IL, USA) as described in Reference [5]. Streptavidin plates preblocked with SuperBlock were purchased from Thermo Scientific (Waltham, MA, USA). Phosphate buffered saline (PBS) was prepared from commercially available tablets from Oxoid Ltd. (Basingstoke, UK). Sybr^®^ Gold was purchased from Invitrogen (Carlsbad, CA, USA).

### 4.2. Assembly of DNA Nanostructures

All DNA nanostructures were assembled by an annealing process using ProFlex PCR System (Applied Biosystems, Foster City, CA, USA). Equal molar amounts of single-stranded DNA at a final concentration of 20 µM were mixed in 1 × PBS. The mixture was first denatured at 95 °C for 5 min and annealed slowly at the rate of −0.5 °C/30 s to 20 °C. The assembled products were stored at 4 °C for long-term storage.

### 4.3. Polyacrylamide Gel Electrophoresis (PAGE) Showing the Formation of DNA Nanostructures

The assembly of DNA nanostructures was confirmed by 12% polyacrylamide gel electrophoresis (PAGE) under 100 V for 1 h. An oligonucleotide concentration of 150 nM was loaded into each lane. The gels were stained by Sybr^®^ Gold and visualized by Gel Doc XR + imager (Bio-Rad, Hercules, CA, USA).

### 4.4. Aptamer-DNA Nanostructure Functionalization of 96-Well Plates

The assembled nanostructures were immobilised onto streptavidin coated 96-well plates using the following steps: the wells were washed three times with phosphate buffered saline with Tween^®^ 20 (PBST) (0.1% Tween-20), then 100 µL of 2 µM biotinylated nanostructure in PBS was added for 2 h incubation, followed by three washes with PBST, and the plate could be used for APTEC or stored at 4 °C for storage.

### 4.5. APTEC Assay Protocol

100 µL of the sample was added to each aptamer-decorated well in triplicate for 1 h, followed by washing with 5 × PBST. A 120 µL amount of the pre-prepared l-lactate/nitrotetrazolium blue chloride (NTB) solution was added and left to incubate for 45 min with mild shaking in the dark. A 100 µL amount of acetic acid (5%) was added to stop the reaction and the absorbance at 570 nm was recorded. For recombinant LDH samples, the binding buffer was 2 × PBS. For PfLDH-spiked whole rat blood samples, 50 µL of the whole blood sample was mixed with 50 µL of PBS (0.5% Triton X-100) to allow for red blood cell lysis prior to incubation.

## Supplementary Materials

The following are available online, Table S1: Sequences of all DNA oligonucleotides for this work, Figure S1: Formation of different DNA polyhedra.
