# Supplementary material for: Aptamer Display on Diverse DNA Polyhedron Supports
Source: Molecules. 2018 Jul 11;23(7):1695. doi: 10.3390/molecules23071695 (PMC6099927; doi:10.3390/molecules23071695)
Supplement: Supplementary file 1 [file molecules-23-01695-s001.pdf]

Supporting Information

Aptamer Display on Diverse DNA Polyhedron Supports

Simon Chi-Chin Shiu [1,†], Lewis A. Fraser [1,†], Yifan Ding [1] and Julian A. Tanner [1,*]

[1] School of Biomedical Sciences, Li Ka Shing Faculty of Medicine, The University of Hong Kong, Pokfulam, Hong Kong SAR China; simon.chichin.shiu@gmail.com (S.C.C.S.); lewis-fraser@hku.hk (L.A.F.); yifand16@gmail.com (Y.D.)

[*] Correspondence: jatanner@hku.hk

[†] These authors contributed equally to this work

Label	Sequence (5’-3’)	Strands required for nanostructures
Ta	biotin-TTTTGCGGCTGGAGCCATGTCATCAGGGGCACGTCTCTAGGACGCGGCCGGGTCT	Tetrahedron 1, 2 and 3
T1b	biotin-TTCATGGCTCCAGCCGCAATGACGGGACCGTGTGCCTGAGC	Tetrahedron 1
T1c	biotin-TTGAGACGTGCCCCTGATGTGCTCAGGCACTGCAGGGCAAA	
T1d	CTGGGCGGTAGAACCATAGTGACCCAGCCGTCTACTTCGGTCCCGTCTAGACCCGGCCGCGTCCTTTTTGCCCTGC	
T2b	biotin-TTCATGGCTCCAGCCGCAATTATCTAGCGATCTCACCTCGTCAAGGGTAAGCCCG	Tetrahedron 2
T2c	biotin-TTGAGACGTGCCCCTGATGTCGGGCTTACCCTTGACGTGTGGTAATTGTTGTGTA	
T2d	CTGGGCGGTAGAACCATAGTGACCCAGCCGTCTACTTGGTGAGATCGCTAGATATAGACCCGGCCGCGTCCTTTACACAACAATTACCAC	
T3b	biotin-TTCATGGCTCCAGCCGCAATTGATCATTGAGTGTCGGTGGCGATGTCTCCCCGATAAAGCGTATGTTATAAGTCT	Tetrahedron 3
T3c	biotin-TTGAGACGTGCCCCTGATGTAGACTTATAACATACGCTTTATCGGGGTCCGGAGCCATCACACCTGGATCAGGAC	
T3d	CTGGGCGGTAGAACCATAGTGACCCAGCCGTCTACTTGACATCGCCACCGACACTCAATGATCATAGACCCGGCCGCGTCCTTGTCCTGATCCAGGTGTGATGGCTCCGG	
C2a	CTGGGCGGTAGAACCATAGTGACCCAGCCGTCTACTTTGGGGCCGGGCGCGCCCCGGTT-biotin	Aptamer with single-stranded and double-stranded spacer
C2b	TTCCGGGGCGCGCCCGGCCCCA	Aptamer with double-stranded spacer
P1a	biotin-TTATGTAACCGTGTCACTCCAGGTGTACTTCCTAAAATCGCGCGACCAG	Square-based pyramid
P1b	biotin-TTCACGGTTACATTCTGTGTTGGCACGGCGTCGTCCGGGTGGCGGTT	
P1c	biotin-TTCACCTGGAGTGTAACCGCCACCCGGACGTCGTTCGTCAAATAGTG	
P1d	biotin-TTTTTTAGGAAGTTCACTATTTGACGAACGTGCTATGCCGCGCGCCT	
P1e	CTGGGCGGTAGAACCATAGTGACCCAGCCGTCTACTTCGCCGTGCCAACACAGTCTGGTCGCGCGTAGGCGCGCGGCATAGC	
P2a	biotin-TTCGGCCGATCTGGCATAGGCTTCCCCAACGTTATCTCGCGTGGCGTACGG	Pentagon-based pyramid
P2b	biotin-TTGATCGGCCGTTCCGCGCGTCCTGCCCTAGACGCCCCTGGGCCC	
P2c	biotin-TTGCCTATGCCTGGGCCCAGGGGCGTCTTGGAGTCAACAGGTCGG	
P2d	biotin-TTCGTTGGGGATCCGACCTGTTGACTCCTCCCGTCGACCAAAGAT	
P2e	biotin-TTCGCGAGATATATCTTTGGTCGACGGGTCTCAGCCCTGACCCCA	
P2f	CTGGGCGGTAGAACCATAGTGACCCAGCCGTCTACTTGGGCAGGACGCGCGGATCCGTACGCCTTGGGGTCAGGGCTGAG	
Pr1	GCAACAGAGCCCTATTCTGTCTCAGTCCACCGGGTTAGGAAACGCGAGTCAGT	
Pr2	CTGGGCGGTAGAACCATAGTGACCCAGCCGTCTACTTCCCTAACGCCTGCGTGAATA	

	GGGCTCTGTTGC	Prism
Pr3	CTGGGCGGTAGAACCATAGTGACCCAGCCGTCTACTTGATCTCAACTCAGTTACCCG GTGGACTGAGAC	
Pr4	CTGGGCGGTAGAACCATAGTGACCCAGCCGTCTACTTTCATCTGGGGCCTTTACTGAC TCGCGTTTCCT	
Pr5	biotin-TAAGGCCCCAGATGATAAACCCAGGCCGGCCCT	
Pr6	TCGCTCGACAGAGATACTATTAGTACCAGGCCCGATAGGGCCGGCCTGGGTTT	
Pr7	biotin-TCGCAGGCGTTAGGGTTCGGGCCTGGTACTAAT	
Pr8	biotin-TACTGAGTTGAGATCTGTATCTCTGTCGAGCGA	
Pr9	CCCTAACGCCTGCGTGAATAGGGCTCTGTTGC	Pr2 without aptamer
Pr10	GATCTCAACTCAGTTACCCGGTGGACTGAGAC	Pr3 without aptamer
Apt	biotin-CTGGGCGGTAGAACCATAGTGACCCAGCCGTCTAC	

Table S1. DNA oligonucleotides samples used in the study.

(a)

Ta		✓				✓	✓	✓
T1b			✓			✓	✓	✓
T1c				✓			✓	✓
T1d					✓			✓
10 bp ladder	✓							

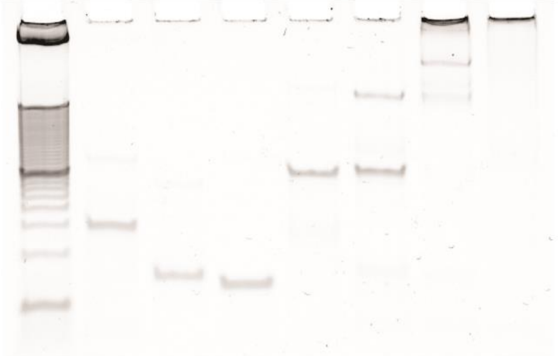

(b)

Ta		✓				✓	✓	✓
T3b			✓			✓	✓	✓
T3c				✓			✓	✓
T3d					✓			✓
10 bp ladder	✓							

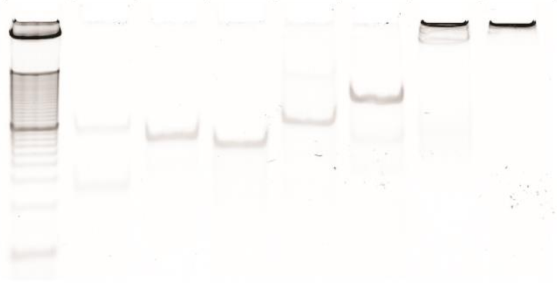

(c)

P1a		✓					✓	✓	✓	✓
P1b			✓				✓	✓	✓	✓
P1c				✓				✓	✓	✓
P1d					✓				✓	✓
P1e						✓				✓
10 bp ladder	✓									

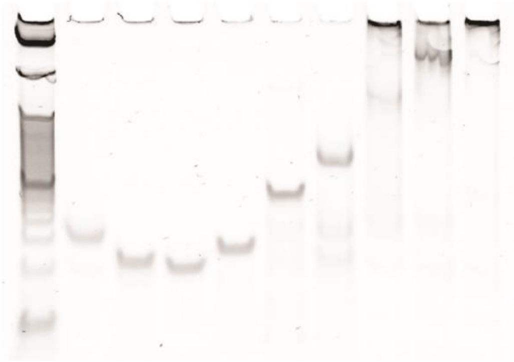

(d)

P2a		✓						✓	✓	✓	✓	✓
P2b			✓					✓	✓	✓	✓	✓
P2c				✓					✓	✓	✓	✓
P2d					✓					✓	✓	✓
P2e						✓					✓	✓
P2f							✓					✓
10 bp ladder	✓											

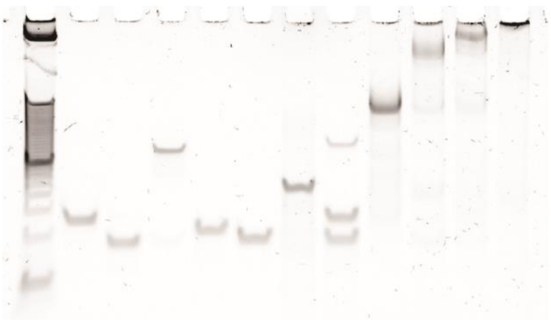

(e)

Pr1		✓							
Pr2			✓						
Pr3				✓					
Pr4					✓				
Pr5						✓			
Pr6							✓		
Pr7								✓	
Pr8									✓
10 bp ladder	✓								

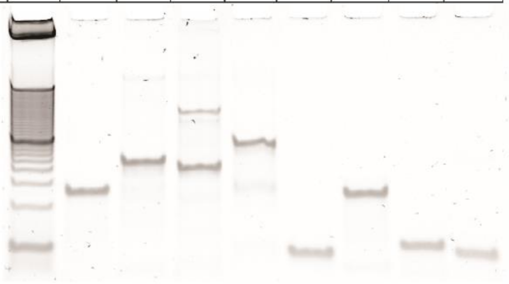

(f)

Pr1		✓	✓	✓	✓	✓	✓	✓
Pr2		✓	✓	✓	✓	✓	✓	✓
Pr3			✓	✓	✓	✓	✓	✓
Pr4				✓	✓	✓	✓	✓
Pr5					✓	✓	✓	✓
Pr6						✓	✓	✓
Pr7							✓	✓
Pr8								✓
10 bp ladder	✓							

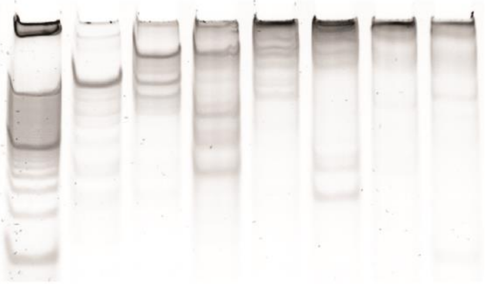

Figure S1. Formation of different DNA polyhedra. 150 nM of DNA was in each lane. **(a)** Tetrahedron 1 of theoretical height 2.65 nm. **(b)** Tetrahedron 3 of theoretical height of 10.55 nm. **(c)** Square-based pyramid. **(d)** Pentagon-based pyramid. **(e)** and **(f)** Formation of DNA prism with three aptamers as in Figure 5(c).
